# The initial learning curve for the ROSA® Knee System can be achieved in 6-11 cases for operative time and has similar 90-day complication rates with improved implant alignment compared to manual instrumentation in total knee arthroplasty

**DOI:** 10.1186/s40634-021-00438-8

**Published:** 2021-12-20

**Authors:** Luc Vanlommel, Enrico Neven, Mike B. Anderson, Liesbeth Bruckers, Jan Truijen

**Affiliations:** 1 Ziekenhuis Oost-Limburg, Strippestraat 20, 3600 Genk, Belgium; 2 Zimmer Biomet, Warsaw, IN USA; 3 Hasselt University, Diepenbeek, Belgium

**Keywords:** Total knee arthroplasty, Robotic surgery, ROSA, Learning curve, Operative times

## Abstract

**Purpose:**

The purpose of this study was to determine the learning curve for total operative time using a novel cutting guide positioning robotic assistant for total knee arthroplasty (raTKA). Additionally, we compared complications and final limb alignment between raTKA and manual TKA (mTKA), as well as accuracy to plan for raTKA cases.

**Methods:**

We performed a retrospective cohort study on a series of patients (*n* = 180) that underwent raTKA (*n* = 90) using the ROSA Total Knee System or mTKA (*n* = 90) by one of three high-volume (> 200 cases per year) orthopaedic surgeons between December 2019 and September 2020, with minimum three-month follow-up. To evaluate the learning curve surgical times and postoperative complications were reviewed.

**Results:**

The cumulative summation analysis for total operative time revealed a change point of 10, 6, and 11 cases for each of three surgeons, suggesting a rapid learning curve. There was a significant difference in total operative times between the learning raTKA and both the mastered raTKA and mTKA groups (*p* = 0.001) for all three surgeons combined. Postoperative complications were minimal in all groups. The proportion of outliers for the final hip-knee-ankle angle compared to planned was 5.2% (3/58) for the mastered raTKA compared to 24.1% (19/79) for mTKA (*p* = 0.003). The absolute mean difference between the validated and planned resections for all angles evaluated was < 1 degree for the mastered raTKA cases.

**Conclusion:**

As the digital age of medicine continues to develop, advanced technologies may disrupt the industry, but should not disrupt the care provided. This cutting guide positioning robotic system can be integrated relatively quickly with a rapid initial learning curve (6-11 cases) for operative times, similar 90-day complication rates, and improved component positioning compared to mTKA. Proficiency of the system requires additional analysis, but it can be expected to improve over time.

**Level of evidence:**

Level III Retrospective Therapeutic Cohort Study.

## Introduction

Introducing new systems into a standardized procedure requires significant adjustment of the surgical workflow for both the surgeon and their team. Recent studies have confirmed improved accuracy and reliability of implant placement and improved early functional recovery in robotic assisted total knee arthroplasty (raTKA) compared to conventional manual total knee arthroplasty (mTKA) [4, 17, 23, 30]. However, controversy exists on whether or not the value of these expensive robotic systems is warranted in the short-term and whether or not the long-term outcomes are improved [10, 11, 14, 21, 25, 27]. Further, efficiency in the operating theatre itself may be affected and warrants review.

The surgeon must decide whether the technology can influence their practice, improve accuracy, and most importantly improve patient outcomes. This is especially important as there is a learning curve associated with the integration of innovative surgical techniques, including raTKA [9, 13, 24, 30]. Naziri et al. [18] recently concluded that the adoption of raTKA by robotic-naïve surgeons is not only feasible, but can be done without concern for increased risks. They also noted comparable surgical times with manual instrumentation after their initial 20 cases. Kayani et al. [9] have reported a learning curve of 7 cases associated with surgical times, but no learning curve in regards to accurate implant placement.

A novel system for raTKA has recently demonstrated accurate component placement using a robotic surgical arm to place and hold cutting guides for bone resection in raTKA [20]. This allows the surgeon to remain in control of the saw, whilst stabilizing and placing the cutting guide with robotic precision. The system provides intraoperative data on the resections and their impact on the soft-tissue envelope. Improved accuracy compared to manual instrumentation in a cadaveric study has been reported [22]. However, clinical data is currently limited to a small case series demonstrating the functionality of the system [1]. As such, further evidence is necessary to evaluate the learning curve and clinical accuracy associated with this robotic system.

The primary aim of this study was to determine the initial learning curve for raTKA through assessments of operative time. Additionally, we reviewed postoperative complications, final limb alignment, and accuracy to plan for the robotic cases.

## Materials and methods

### Patient population

**Fig. 1:**
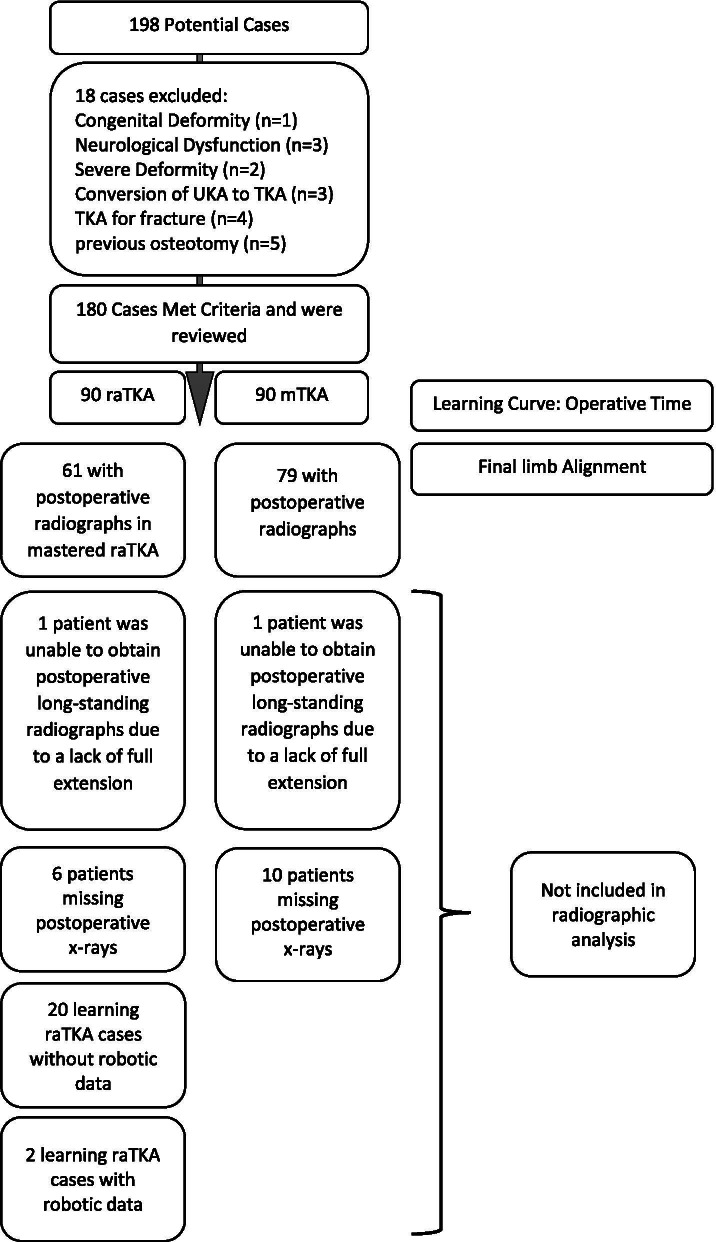
Flow chart demonstrating patient attrition

After receiving approval from the ethics review board (Ziekenhuis Oost Limburg Genk - CTU2020022) a retrospective cohort study on a series raTKA or mTKA cases performed by one of three high-volume (> 200 cases per year) arthroplasty surgeons was conducted. The raTKA cases included the first 30 consecutive patients for each operating surgeon (*n* = 90) that met all inclusion criteria and none of the exclusion criteria (below). The mTKA group consisted of a consecutive series of 30 cases for each surgeon that occurred concurrently with the raTKA cases (*n* = 90). All patients provided informed consent. Patients were allocated to either raTKA or mTKA based on availability of the robotic device on the date of their surgery. All cases were performed between December 2019 and September 2020. Patients were included in the study if they were between the ages of 18 and 80 years, had an indication for primary TKA due to osteoarthritis, and had surgery by one of the surgeon authors (LV, EN, JT). Patients with congenital deformity, underlying neurological dysfunction, severe deformity (> 15 degrees of preoperative varus/valgus alignment or a non-correctable deformity), a prior infection or osteotomy around the knee, prior unicompartmental procedure or osteotomy, or fracture as the primary indication were excluded (*n* = 18). No cases were excluded due to conversion to mTKA for mechanical failures. Ultimately, 180 patients (Fig. 1) were included, with 60 (30 raTKA and 30 mTKA) patients from each surgeon. To further evaluate the learning curve, the raTKA group was split into consecutive groups of 10.The STROBE guidelines were followed [28].

### Surgical technique and recovery

Surgeons underwent cadaveric training on the robotic system (ROSA® Knee System, Zimmer Biomet, Montreal, Quebec, Canada) prior to performing the procedure in the operating theatre. The surgeons also provided support amongst themselves by assisting each other on the first two cases, before performing the next cases on their own. Two surgeons had prior experience with other robotic systems, but this was minimal < 20 cases for one using a robotic-arm assisted surgery for partial knee arthroplasty and < 10 cases for the other using a hand-held burring robotic device. None of the surgeons had prior experience with other computer assisted surgery technologies. The same surgical technique with the subvastus approach, extension gap first, was used in all cases. Additionally, all patients received a patellar resurfacing. Every case for all surgeons included the same operating staff with one assisting nurse, a resident, and the same industry representative for all surgeons. The same prosthesis design (Persona® Posterior Stabilized; Zimmer Biomet, Warsaw) was used in all cases.

**Fig. 2:**
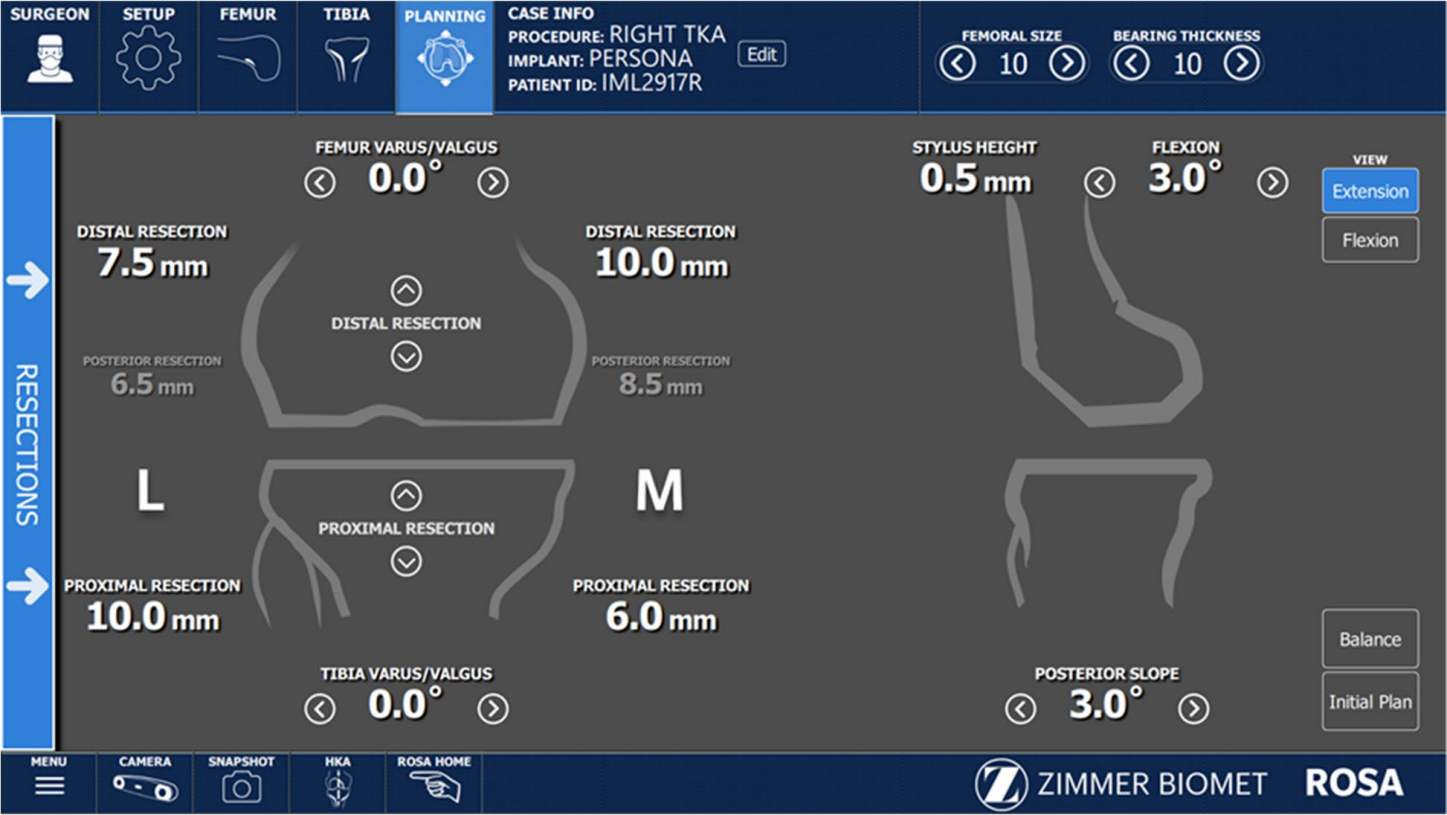
An intraoperative screen shot from the robotic system demonstrating the surgeon’s ability to plan resection depths and component sizes

**Fig. 3:**
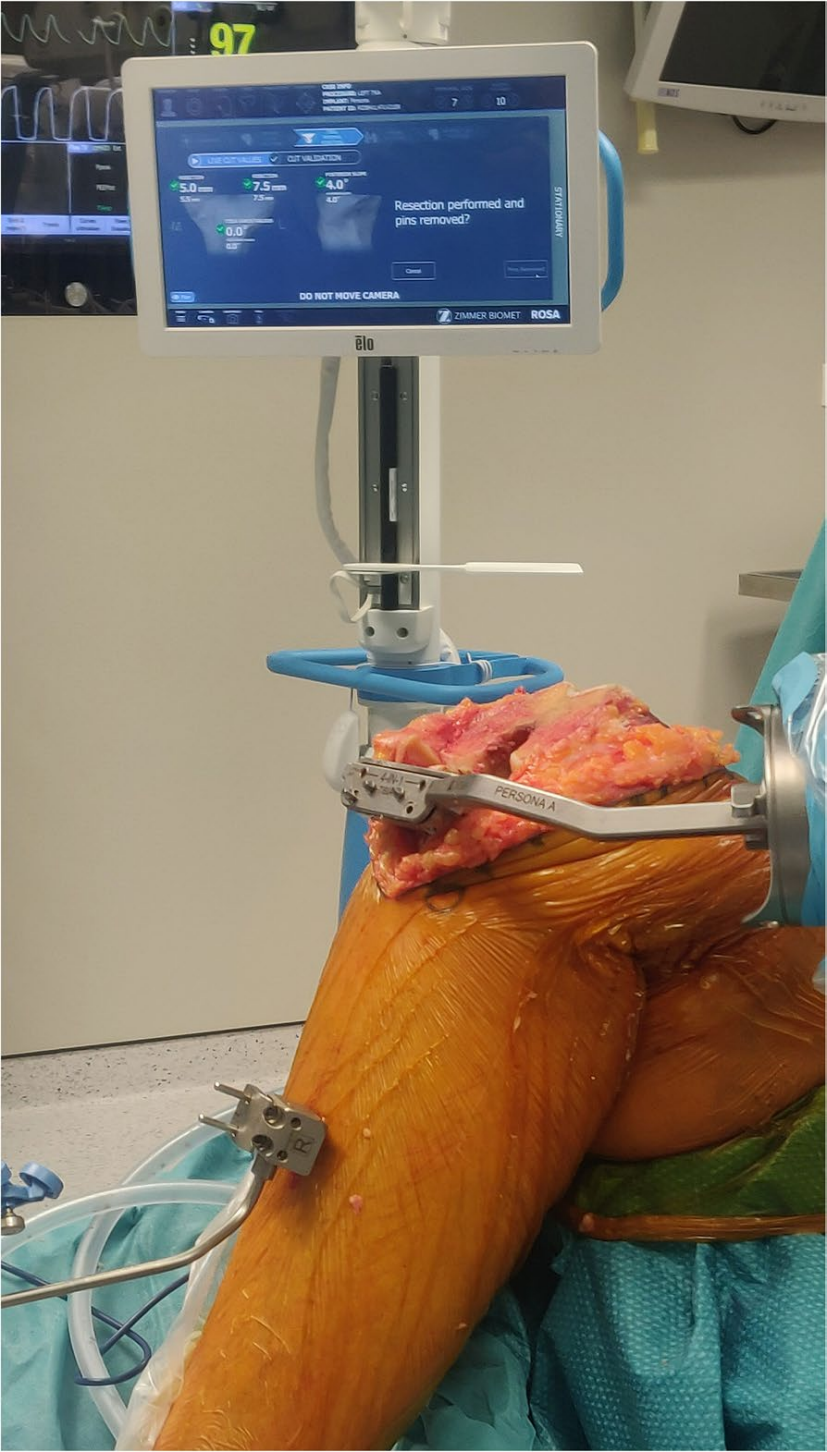
An intraoperative image demonstrating the cut guide positioned by the robotic arm

**Fig. 4:**
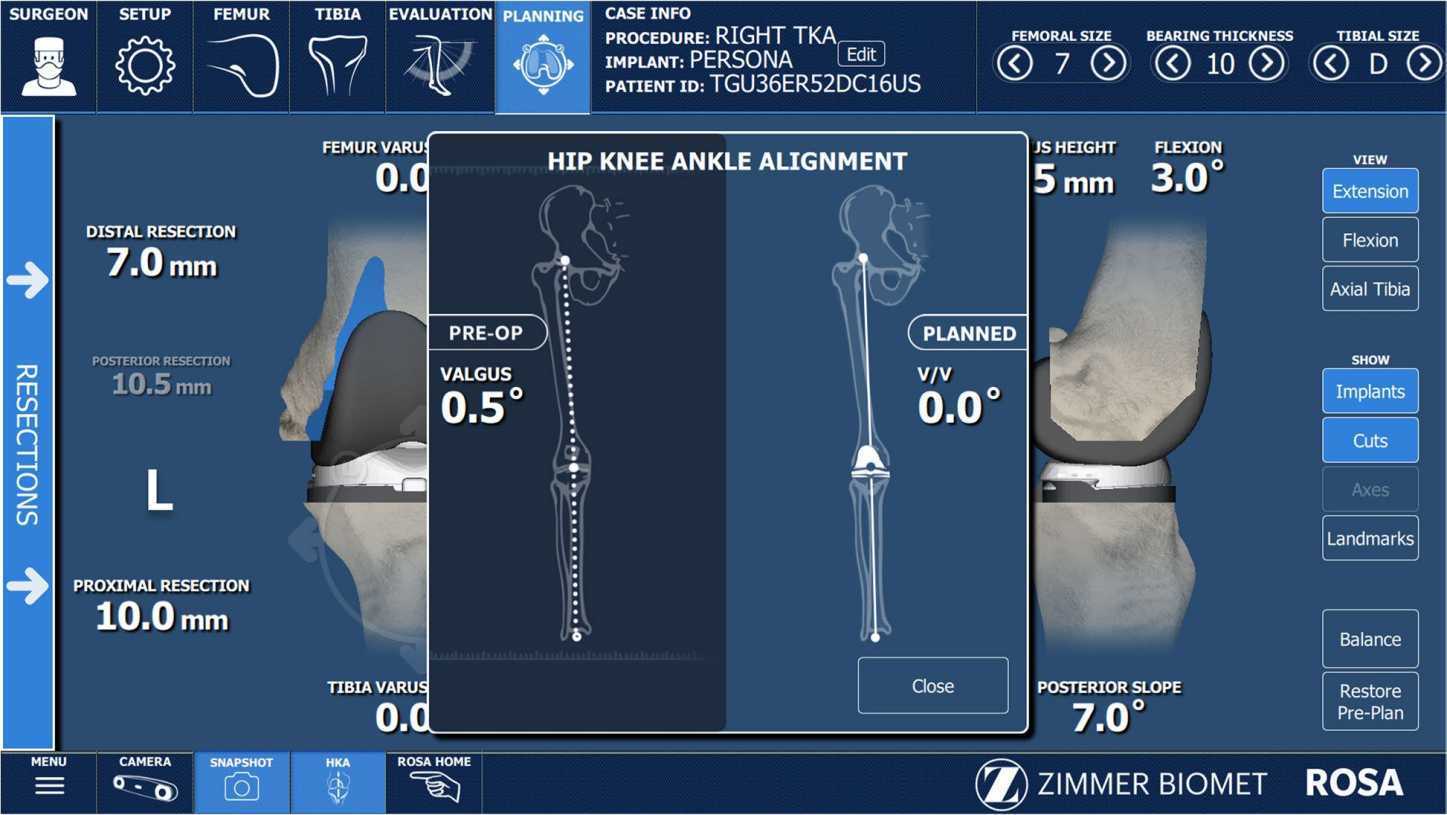
An intraoperative image demonstrating the alignment assessment by the robotic system

**Fig. 5:**
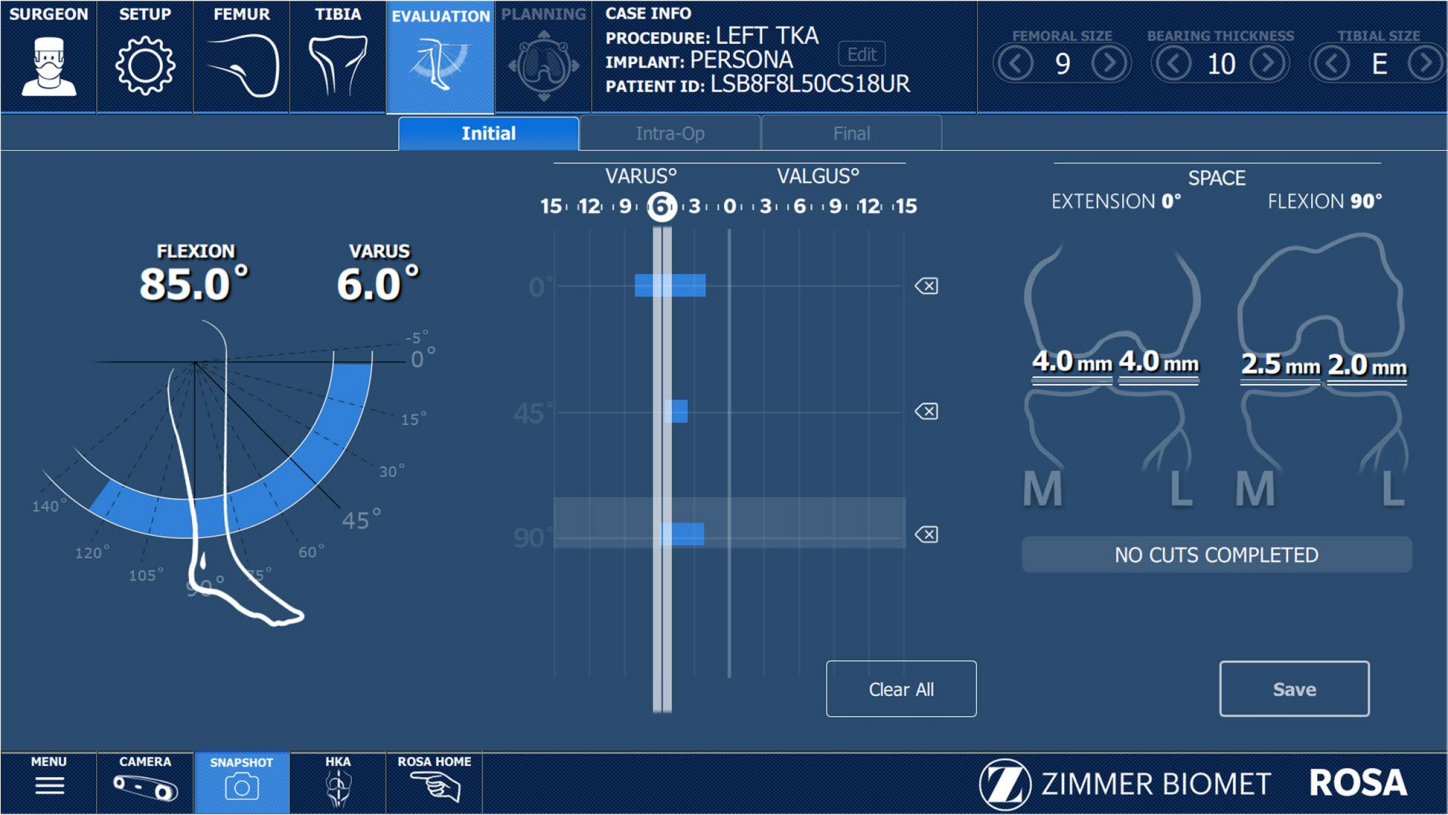
An intraoperative image demonstrating the assessment of range of motion and ligament laxity both medially and laterally by the robotic system

All raTKA cases were performed image-free using intraoperative data to achieve the preferred component placement and size (Fig. 2). Distal femoral and proximal tibial bi-cortical registration pins were inserted, and fixed optical trackers mounted. Bone registration was performed using boney landmarks displayed on the computer screen to verify anatomy and establish bone geometry. Joint balancing captured femoral and tibial poses with corrective forces, assessed kinematics through the arc of motion, and enabled fine tuning of implant positioning based on laxity of the soft tissue envelope. A robotic arm was used to execute the perioperative plan by placing the cutting jig in the appropriate plane, removing the need for traditional intramedullary guides (Fig. 3). Tibial and femoral osteotomies in the coronal plane were performed to achieve the overall alignment as desired by the surgeon (Fig. 3). In the sagittal plane, 3°- 5° of femoral component flexion were used to optimize implant sizing whilst preventing notching. The tibial slope was initially set to 4.5 degrees for the initial cut and then adjusted as required based on intra- operative assessment of the flexion gap (Zimmer FuZion® Tensor, Zimmer Biomet, Warsaw, IN, USA) and range of motion (ROM). Optical motion capture technology was used to assess limb alignment (Fig. 4), ROM (Fig. 5), flexion and extension gaps (Fig. 5), and arc of motion with trial implants prior to definitive selection and cement implantation (Refobacin® R, Zimmer Biomet, Warsaw, IN, USA).

Conventional mTKA was performed using standard instrumentation and all were fully cemented (Refobacin R, Zimmer Biomet, Warsaw, IN, USA). Intramedullary referencing was used to perform tibial and femoral bone resections. Flexion and extension gaps were checked using the same tensor device as raTKA and appropriate soft tissue releases performed to ensure balanced gaps. No further intraoperative adjustments or tailoring of implant positioning were performed to account for individual patient anatomy.

Every patient followed the same postoperative protocol, bed rest the day of surgery. Bed rest at day one with twice a day continuous passive motion (CPM) for 45-60 min. On day two, mobilization with crutches or a walker was added. Ascending/descending stairs with aid was added on day three. If pain was under control, wound was dry, knee flexion was over 60° and patients were self-sustainable with crutches, they could leave the hospital at postoperative day three or four.

### Data collection and analysis

As some studies have reported learning curves in raTKA for surgical time between 20 and 40 cases, we included the first 30 raTKA cases with their concurrent mTKA cases for each surgeon as a convenience sample [18, 26]. Due to the limited follow-up time (minimum three-month) no further adjustments were made to the sample size. Due to geographical or logistical reasons some patients lacked postoperative radiographs (Fig. 1). Additionally, two patients lacked full extension (*n* = 1 for raTKA and mTKA) at 6 weeks postoperatively and were unable to obtain the full-length hip-knee-ankle (HKA) radiographs.

The learning curve for raTKA was assessed according to the recommendations of Hopper et al. [7] where both measures of surgical performance and early patient outcomes were assessed. Chart reviews were performed to obtain operative times for the surgical performance and 90-day postoperative complications to assess early patient outcomes. Radiographs were reviewed to assess final limb alignment (HKA angle) and planned vs. validated measures in the raTKA cases were also reviewed.

Operative time was defined as time from initial surgical incision to final wound closure. In raTKA, surgical times for the following parts of the procedure were also recorded: set-up (surgical tray, robotic device, and instruments), registration (surgical approach, insertion, registration of pins and bone registration), joint balancing, bone preparation, implant trialing, cement implantation of final prosthesis, and overall operative time.

Accuracy of achieving the planned alignment of the leg was determined based on full-length HKA radiographs. They were evaluated according to methods of Cooke et al. [6]. The hip center was obtained using concentric Mose circles. The goal for mTKA was to achieve zero degrees for the HKA angle, resulting in a neutral mechanical alignment (MA). However, this goal was adjusted in some cases with more severe deformity resulting in cases that were under corrected (not planned nor achieved a neutral mechanical alignment). Radiographic outliers for mTKA were considered as patients whose mechanical alignment was > ±3° of planned neutral MA. In raTKA, outliers were defined as being > ±3°of the intraoperative planned HKA angle. This was due to the surgeon’s using an adjusted mechanical alignment in raTKA to minimize ligamentous releases.

Implant positions were assessed as described by Moon et al. [15]. The femoral coronal implant alignment was measured as the lateral angle subtended by the femoral mechanical axis and the line connecting the distal points of the medial and lateral condyles of the femoral component known as the distal lateral femoral angle (DLFA). The femoral sagittal implant alignment (femoral flexion angle) was calculated as the angle subtended between the perpendicular line running proximally from the distal femoral surface in contact with the femoral component and the femoral mechanical axis. The tibial coronal implant alignment was measured as the medial angle subtended by the tibial mechanical axis and the medial to lateral axis of the tibial implant known as the medial proximal tibial angle (MPTA). The tibial sagittal alignment (tibial slope) was calculated as the angle between the tibial mechanical axis and anterior to posterior axis of the tibial implant. Anteroposterior radiographs were used to measure the joint line height by calculating the perpendicular distance from a line extending through the distal points of the femoral condyles and a parallel line extending to the fibular head. True lateral knee radiographs were used to calculate the posterior tibial slope [2] and posterior condylar offset ratio (PCOR) [8].

The radiological measurements were reviewed by two independent observers and MA was assessed for interrater agreement. Each observer was blinded to the other’s measures. The average of the two was used as the final measure. When looking at interrater agreement for mechanical alignment, a Bland-Altman plot demonstrated good agreement around the zero bias line and within the 95% confidence interval.

The cumulative summation sequential analysis tool (cumsum) [5, 29] was used to assess the learning curves in raTKA for operative time. Standardized target values for the cumsum analyses were set using the (surgeon-specific) mean values for the overall operative time from the raTKA group. Cumsum values represent a running total of the differences between the value of each data point and the standardized target. To determine the point at which the learning phase was over, we used a piecewise linear regression model. This model describes the cumsum function as two lines and the point where the lines connect (‘change point’) is the transition between learning phase and post-learning phase (mastered).

Continuous variables are presented by means and standard deviations. The difference between the planned and validated angles are reported as absolute values. Counts and percentages are used for categorical data. The raTKA and mTKA groups were compared with respect to patient characteristics using the chi square test and Fisher’s exact test for categorical data; and by means of an independent t test or Mann–Whitney test for continuous variables.

The mTKA, learning raTKA, and mastered raTKA groups were compared for operative time by means of two-way ANOVA models. To correct for a possible surgeon effect the ANOVA models included, both a group (mTKA, learning raTKA, and master raTKA) effect and a surgeon effect. Least-square means and 95% confidence intervals obtained for this model are presented. Statistical significance was set at *p* < 0.05 for all statistical tests. Analysis was performed using SAS for Windows software version 9.4 by an independent statistician (LB).

## Results

Both groups were checked for baseline demographics, and there was no difference in demographics between the raTKA and mTKA groups (Table 1). There was also no difference in preoperative varus/valgus deformity between groups (*p* = 0.966, Table 1). Similarly, there was no difference in patient demographics between the consecutive groups of ten (Table 2.)

**Table 1:** Patient characteristics for entire population, all three surgeons combined

Characteristic	raTKA, *n* = 90	mTKA, *n* = 90	*P* value
Age (years), mean (SD, range)	68.7 (8.1, 46 – 84)	69.8 (8.2, 47 – 86)	0.358
BMI, mean (SD, range)	31.32 (5.20, 24 – 48.4)	30.49 (4.80, 22.1 – 43.9)	0.267
ASA Score, n (%)			
1	7 (8)	11 (13)	0.091
2	45 (51)	53 (61)	
3	37 (42)	23 (26)	
Female Sex, n (%)	44 (49)	46 (52)	0.652
Pre-operative Mechanical Alignment (median, range)	5 (−13 – 23)	5 (−10 – 18)	0.966

*BMI* body mass index (kg/m^2^)

**Table 2:** Patient characteristics for raTKA groups of ten, all three surgeons combined

Characteristic	1 – 10, *n* = 30	11 – 20, *n* = 30	21 – 30, *n* = 30	*P* value
Age (years), mean (SD)	67 (6.2)	71 (9.1)	68 (8.5)	0.209
BMI, mean (SD)	30.98 (4.20)	31.11 (5.48)	31.88 (5.90)	0.775
ASA Score, n (%)				
1	2 (7)	4 (13)	1 (3)	0.066
2	16 (55)	9 (30)	20 (67)	
3	11 (38)	17 (57)	9 (30)	
Female Sex, n (%)	15 (50)	13 (43)	16 (53)	0.733

**Fig. 6:**
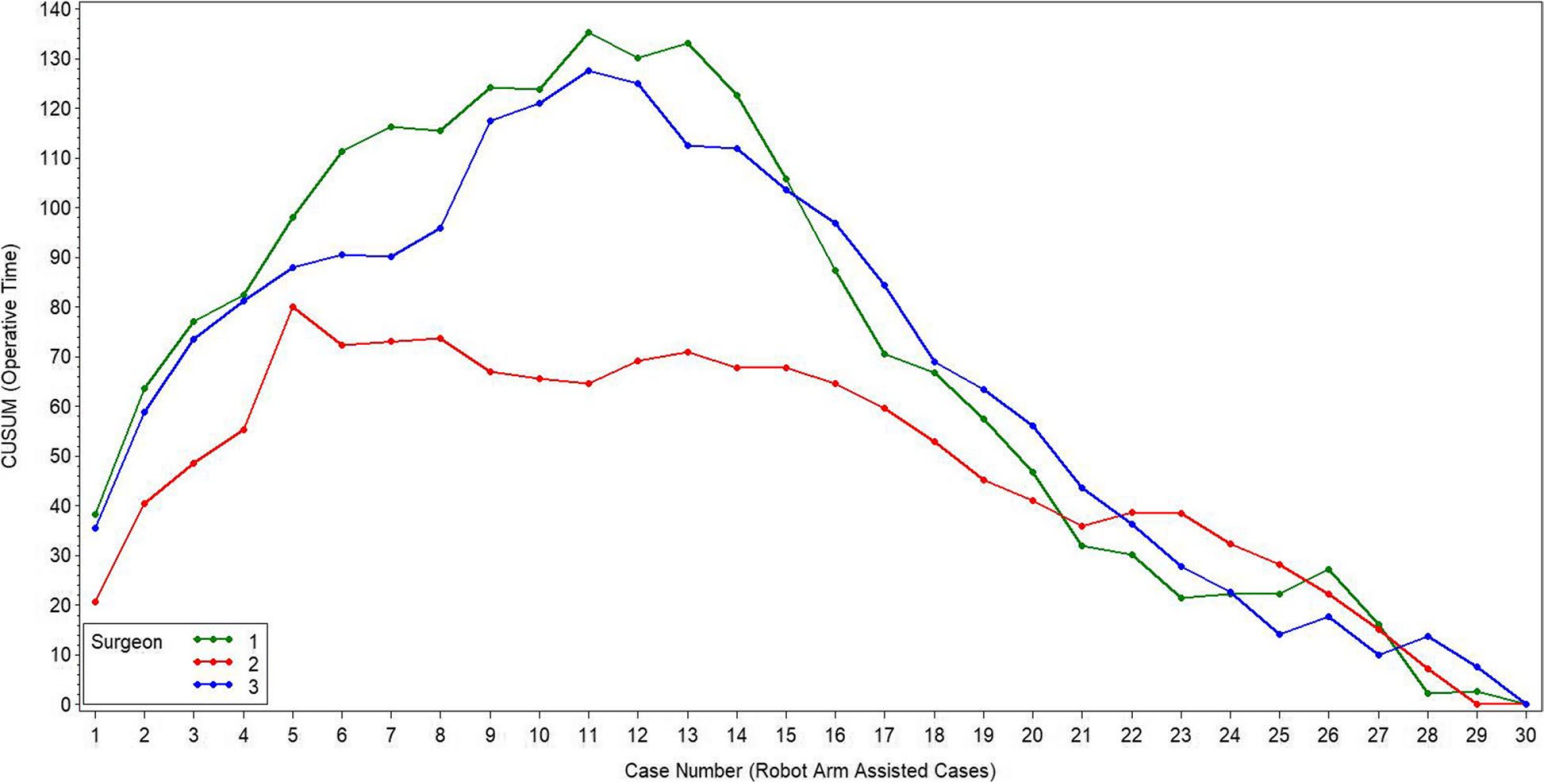
The cumsum figure shows the cumulative sum of the differences of the operating times compared to the average operating time for raTKA for each of the surgeon’s cases

**Fig. 7:**
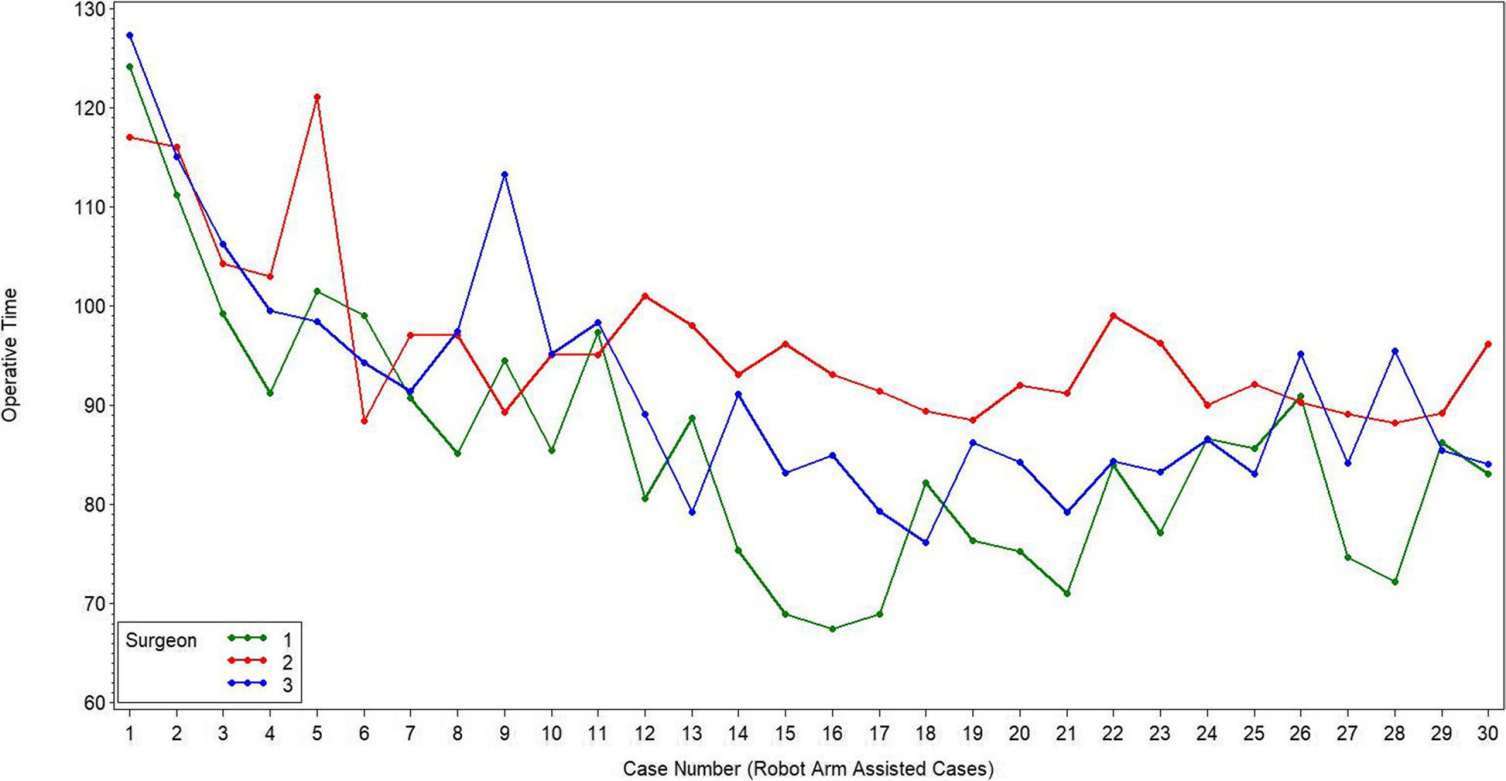
This line graph demonstrates the trend of surgical times across cases per surgeon

The initial learning curve for total surgical time was 10 cases for surgeon one, six cases for surgeon two, and 11 cases for surgeon three (Fig. 6). There was a difference in total operative time between the learning raTKA and both the mastered raTKA and mTKA groups (both, *p* = 0.001, Table 3, Fig. 7) for all three surgeons combined. The mastered raTKA group was also significantly longer (p = 0.001) than the mTKA by a least mean square difference of 13.17 min (standard error 1.61 min). The average overall surgical time for raTKA was greater in the first 10 cases for all three surgeons combined (Table 4, *p* < 0.001). There was a significant reduction in times for the robotic setup, bone registration, joint balancing, bone preparation, and implant trialing between the first ten and final ten cases (Table 4). The average surgical time for all surgeons was 91.3 ± 11.9 min in raTKA cases versus 73.3 ± 11.08 min in mTKA (p < 0.001).

**Table 3:** Total operative time (minutes) between the learning raTKA, mastered raTKA, and mTKA groups. Reported as least square (LS) mean and standard error (SE)

Comparison	LS mean (Std err.)	95% CI	*P* Value
Learning	102.40 (1.89)	[98.68; 106.12]	
Mastered	86.51 (1.24)	[84.07; 88.95]	
mTKA	73.34 (1.03)	[71.30; 75.38]	
**Difference**			
Learning vs. Mastered	15.89 (2.26)	[11.44;20.34]	0.001
Learning vs. mTKA	29.06 (2.15)	[24.81; 33.31]	0.001
Mastered vs. mTKA	13.17 (1.61)	[9.99;16.35]	0.001

**Table 4:** Comparisons of operating room times (minutes) between raTKA groups of ten, all three surgeon’s cases combined, reported as mean (SD)

Variable	1 – 10, *n* = 30	11 – 20, *n* = 30	21 – 30, *n* = 30	*P* value
Surgical Time	101.6 (11.6)	85.7 (9.4)	86.48 (6.94)	< 0.001
Robotic Setup Time	8.9 (1.9)	7.5 (1.1)	7.3 (0.80)	<.0001
Surgical Approach Time	9.5 (1.7)	8.9 (1.5)	8.6 (1.3)	0.074
Bone Registration	10.3 (2.0)	8.2 (1.7)	7.5 (1.4)	< 0.001
Joint balancing	7.0 (1.9)	3.8 (0.7)	4.1 (1.4)	< 0.001
Bone preparation	28.8 (6.0)	25.7 (3.1)	26.4 (3.5)	0.020
Implant Trialing	17.3 (4.0)	14.9 (4.3)	13.5 (2.7)	< 0.001

There were no intraoperative or postoperative complications associated with the robotic system. Postoperative complications were minimal and included arthrofibrosis (learning raTKA = 1, mastered raTKA = 1, and mTKA = 1), surgical site infections (SSI, mastered raTKA = 1, mTKA = 3), deep vein thrombosis (DVT, mastered raTKA = 1, mTKA = 0), and periprosthetic joint infection (PJI, raTKA = 0, mTKA = 1). Only one of the SSIs (mTKA) required intervention consisting of wound revision. For the three arthrofibrosis cases, one (mastered raTKA) was diagnosed at 12 weeks and had manipulation under anesthesia (MUA), with good results. The other two (one each mTKA and learning raTKA) were diagnosed at 6 weeks and received oral steroids for 4 weeks with improvement of their function, avoiding MUA. The one PJI case underwent polyethylene exchange 3.5 months after the index procedure associated with severe erysipelas.

There was a difference in the frequency of radiographic outliers between mastered raTKA cases and mTKA cases (*p* = 0.003). The proportion of outliers was 5.2% (3/58) for the mastered raTKA compared to 24.1% (19/79) for mTKA cases reviewed. There was no difference in postoperative MA between the raTKA groups of 10 (*p* = 0.602). The mean ± standard deviation for each group was 1.3° ± 2.03° for group one, 1.6° ± 1.5° for group two, and 1.1° ± 2.3° for group three.

For the mastered raTKA cases we assessed the difference between the validated and planned angles for the DLFA, MPTA, femoral flexion, and tibial slope. The absolute mean difference for all angles was < 1 degree (Table 5). As there were only 2 cases in the learning cases that had both the planned and validated values saved, the learning cases were excluded from this analysis (Fig. 1).

**Table 5:** Comparisons between the validated and planned (validated – planned) angles for the mastered raTKA group. Reported as absolute means

Angle	Mean ± SD	Min - Max
DMFA	0.32 ± 0.25	0 – 1.1
MPTA	0.46 ± 0.32	0 – 1.4
Femoral Flexion	0.40 ± 0.34	0 – 1.4
Tibial Slope	0.89 ± 0.74	0 – 4.4

## Discussion

The main finding of this present work was that the initial learning curve for operative times when adopting this robotic system was 6-11 cases. Postoperative complications were minimal in all groups. Of significance was the greater proportion of cases within the target range of the planned component placement in the mastered raTKA group compared to mTKA.

Previous studies have reported learning curves ranging from 7 to 43 cases [3, 9, 12, 18, 24] with various robotic designs. Using a haptic guided robotic-arm, Kayani et al. [9] described a learning curve of seven cases for operative times using a cumulative summation analysis. As both their system and the one under review employ the use of an optically guided robotic-arm, it seems reasonable that the learning curves should approximate each other, despite some differences in design. When evaluating operative time points within raTKA, there are apparent differences between the robotic system employed in this study and that reported by Kayani et al. [9] They report robotic setup times of 14 ± 4.3 min in their first ten cases which reduced to 8.9 ± 1.2 in their third set of cases (21-30). Whereas, our data showed an initial setup time of 8.9 ± 1.9 min in the first ten cases and 7.3 ± 0.8 min in the third set. They also noted greater registration times than our data. However, bone preparation and implant trialing appear greater with the system used in this report. It is unknown why these differences exist, but they are likely multifactorial. For one, all cases in this study were performed free of pre-operative imaging, thus additional time was used intraoperatively to determine the planned cuts, gaps, rotation, and implant size, which are all part of the balancing, bone preparation and trialing phases of the procedure. Additionally, a tensor device was used to test flexion and extension gaps which were then captured on the system to further guide the procedure. Given the difference in setup up between the robots and surgeon variability it is difficult to compare between robotic systems. Mahure et al. [12] assessed the learning curve using an active robotic system in a multicenter study and reported an initial learning curve 0, 12, 16, and 19 cases using cumulative summations for each of the four surgeons evaluated. Another report noted the learning curve was achieved in the first 20 cases when comparing an initial group of 20 to a subsequent group of 20 haptic guided robotic arm cases [18]. As that study was limited to a univariate comparison between groups further precision on the learning curve could not be elucidated. When assessing the number of cases needed to implement a handheld robotic system into their practice, Bell et al. reported that 29 cases were needed for the surgical time to fall within the range of the mean time. They also noted that the review of the intraoperative plan experienced the greatest reduction in time. This suggests that surgeon confidence in the intraoperative planning for that system improved with time, which is consistent with our continued experience.

We began our experience as a quest to optimize every individual step to reduce operating theatre time. Initially serial steps were used to ensure complete understanding of the process. Next, we sought to identify a smooth algorithm using the robotic system that fit into our natural workflow. We found that having the resident install the trackers during the surgical approach provided some improved efficiency by combining steps. Each step was assessed for efficiency adjustments within each surgeon’s workflow, but no steps were skipped. After this initial learning curve, we continue to see improved proficiency with this system. Further, we feel that with time our confidence with the system and reduced suspicion of error warnings have resulted in more efficiency without sacrificing the precision provided by the robot. This supports our philosophy of adjusted mechanical alignment as we now require less time to evaluate and adjust our surgical plan. As this data represent our initial learning curve, it seems reasonable that continued use of the system will result in improved confidence and efficiency as time goes on. Sodhi et al. [24] have recently suggested time neutrality between mTKA and raTKA can be achieved within several months. When evaluating the surgical time of robotic cases over the course of a year, Marchand et al. [13] reported decreasing surgical times from 81 min at 1 month, to 65 at 6 months and 62 at 1 year. They note that though the innovative technology initially increased operative times, the continued use of said technology resulted in comparable operative times to manual instrumentation. We did not see this affect in our consecutive cases of 10 as cases 11-20 had similar times as 21-30. This is likely due to these cases still being in the early phases of a subsequent proficiency period. However, we have noticed continued reductions in surgical time, anecdotally, with current surgical times being consistent with our mTKA cases.

The adoption of innovative technology may pose increased risk for adverse events following primary TKA and as such, outcomes associated with safety should be included in a learning curve analysis. The occurrence of postoperative complications was minimal in all groups and consistent with rates reported in the literature [16, 19].

The data support the use of this robotic technology to improve implant positioning in primary TKA. The mTKA group experienced a substantial and significant deviation from the planned mechanical alignment (24%) compared the mastered raTKA group (5.4%). This supports previous findings of little if any learning curves associated with the accuracy of component placement with raTKA [9].

As some patients had the option to move to mTKA due to pain or other personal reasons, it is possible that a selection bias exists. However, as patients were enrolled consecutively for each group and concurrently between groups, we feel this potential bias has been minimized. It should also be emphasized that because of this, both groups followed the same care pathways. Further, though it cannot account for all potential sources of selection bias, we also found no difference in patient demographics between the treatment groups, and the consecutive raTKA cases (groups of ten). Unfortunately, we were not able to assess other preoperative measures between the groups and thus this data may suffer from some additional selection bias associated with more complex patients. However, despite not having the grade of osteoarthritis or preoperative range of motion, we do have a comparison of the preoperative deformity (Table 1), and there was no difference in the varus/valgus deformities between groups. Finally, patients in both groups were required to meet the same inclusion and exclusion criteria, and no patients were lost to follow-up. The population is representative of three separate surgeons which may introduce surgeon specific bias in operative times and surgical techniques. However, when evaluating outcomes, a model for the three surgeons together indicated that the differences between the three groups (learning raTKA, mastered raTKA, and mTKA) were not surgeon specific (not from a statistical point of view). Additionally, the same implants, cement, and surgical techniques were followed by each surgeon. Regression models were used to control for potential differences between surgeons. The study is also limited by the potential error associated with radiographic review from postoperative plain film radiographs when assessing component placement accuracy and limb alignment. Despite the risk of error, our data demonstrated good interrater agreement and therefore any errors in measurement calculations should be consistent between the groups. Though we would have liked to evaluate the accuracy in the learning raTKA cases, the data was not captured on the robotic system and thus we are unable to evaluate that group. Patients were allocated to either raTKA or mTKA based on availability of the robotic device on the date of their surgery. As no more than three raTKA cases were able to be performed each surgery day, due to hospital restrictions associated with only 3 instrument sets available, the waiting list for raTKA was delayed and some patients asked for mTKA to avoid further delays. Thus, the patients were enrolled concurrently in each arm and consecutively per arm. Finally, we did not evaluate the learning curve of the surgical support staff, which could affect the efficiency of the robotic implementation. That said, the same industry representative was available for all cases despite the variances in the assisting nurse and resident between and within surgeons. Though the effect on the learning curve by the surgical staff could be present, it is likely minimal between surgeons.

## Conclusion

As the digital age of medicine continues to develop, advanced technologies may disrupt the industry, but should not disrupt the care provided. This cutting guide positioning robotic system can be integrated relatively quickly with a rapid initial learning curve (6-11 cases) for operative times, similar 90-day complication rates, and improved component positioning compared to mTKA. Proficiency of the system requires additional analysis, but it can be expected to improve over time.

## Data Availability

The data is protected medical information, and requests for access to de-identified data will be considered per appropriate requests.
